# The Bascule/Pendular Maneuver: A Novel Repositioning Strategy for the Apogeotropic Variant of Posterior Canal BPPV

**DOI:** 10.3390/audiolres16010023

**Published:** 2026-02-03

**Authors:** Giacinto Asprella-Libonati, Fernanda Asprella-Libonati, Giuseppe Lapacciana, Camilla Gallipoli, Giuseppe Gagliardi, Anna Guida, Giada Cavallaro

**Affiliations:** 1 Otolaryngology Unit, Madonna Delle Grazie Hospital of Matera, 75100 Matera, Italy; giuseppe.lapacciana@asmbasilicata.it (G.L.); camilla.gallipoli@asmbasilicata.it (C.G.); giuseppe.gagliardi@asmbasilicata.it (G.G.); annac.guida@asmbasilicata.it (A.G.); giada.cavallaro@asmbasilicata.it (G.C.); 2 Otolaryngology Unit, Engles Profili Hospital, AST Ancona, 60044 Fabriano, Italy

**Keywords:** benign paroxysmal positional vertigo, posterior semicircular canal, Bascule/Pendular maneuver

## Abstract

Background: Benign paroxysmal positional vertigo (BPPV) is the most common peripheral vestibular disorder and most frequently involves the posterior semicircular canal (PSC). Atypical apogeotropic variants of PSC-BPPV may present with pure down-beating positional nystagmus, mimicking contralateral anterior semicircular canal involvement and resulting in diagnostic and therapeutic uncertainty. Objective: To assess the effectiveness of the Bascule/Pendular maneuver in managing patients with pure down-beating positional nystagmus and suspected apogeotropic PSC-BPPV. Methods: A total of 178 patients presenting with pure down-beating positional nystagmus without a torsional component were evaluated using a standardized diagnostic protocol under video-Frenzel goggle monitoring. All patients underwent the Bascule/Pendular maneuver, a modification of the classical Semont maneuver designed to mobilize otoconial debris along the vertical canal planes (Left Anterior–Right Posterior and Right Anterior–Left Posterior), regardless of precise lateralization. Conversion of nystagmus from the apogeotropic to the geotropic variant was considered the primary outcome. Results: The maneuver was well tolerated, with no procedural interruptions or complications. Immediate conversion to the geotropic variant was achieved in 86 patients (48.3%) after a single maneuver. In the remaining patients, successful conversion was obtained after additional maneuvers, most commonly following a second application on the contralateral plane. Once geotropization was achieved, all patients were successfully treated using a standard posterior canal repositioning maneuver. Conclusions: The Bascule/Pendular maneuver is a practical and effective approach for patients presenting with pure down-beating positional nystagmus and suspected apogeotropic PSC-BPPV. By facilitating conversion to the geotropic form, it allows prompt treatment with conventional repositioning maneuvers and may represent a useful first-line strategy in atypical BPPV presentations.

## 1. Introduction

Benign paroxysmal positional vertigo (BPPV) is recognized as the most common peripheral vestibular disorder, with significant implications for patient’ quality of life due to recurrent episodes of vertigo and imbalance [1,2].

Despite extensive study, the precise pathophysiological mechanisms underlying BPPV remain incompletely understood. It is generally attributed to the detachment of otoconia from the utricular macula, leading to abnormal stimulation of the semicircular canals during head movements [3].

In addition, Akkuzu et al. [4] and Gacek et al. [5] have suggested that degenerative changes in the saccular macula may contribute to the development of BPPV, indicating a broader spectrum of otolithic pathology. Dislodged otoconia may either adhere to the cupula, rendering it sensitive to gravitational forces (cupulolithiasis), or conglomerate and float freely within a semicircular canal, most commonly the posterior canal (canalolithiasis) [6].

The posterior semicircular canal (PSC) variant represents the most frequent form of BPPV, followed by lateral semicircular canal (LSC) involvement, whereas anterior semicircular canal (ASC) variants are rare [7]. Paroxysmal positional nystagmus (PPN), typically observed during diagnostic maneuvers, usually displays characteristic features that allow the clinician to localize the affected canal and infer the direction of otoconial movement. Nevertheless, interpretation may be challenging, as PPN can occasionally mimic involvement of a different semicircular canal [8,9,10,11].

In typical PSC-BPPV, otoconial debris within the ampullary arm generates a torsional up-beating nystagmus (TUB-PPN) as a consequence of the Dix-Hallpike maneuver. This nystagmus consists of a vertical upward component and a torsional component beating toward the undermost ear. The PSC PPN is geotropic, with the torsional component directed toward the ground during both right and left Dix-Hallpike maneuvers [12].

As reported by Vannucchi et al. [13], a variant of posterior semicircular canal BPPV (PSC-BPPV) may present during the Dix–Hallpike maneuver with a torsional down-beating positional nystagmus (TDB-PPN). This pattern is characterized by a vertical component beating downward toward the chin and a torsional component directed toward the uppermost ear relative to the superior corneal pole. Such a nystagmus is defined as apogeotropic, as it is oriented away from the ground in the provoking positions. In this context, the authors hypothesized that posterior semicircular canal (PSC) canalolithiasis, caused by free-floating otoconial debris located near the common crus, may mimic contralateral anterior semicircular canal (ASC) BPPV, as it can produce a similar down-beating nystagmus pattern.

To address this atypical presentation, Asprella et al. [14] described two specific repositioning techniques—the rapid Demi Semont maneuver and the static 45° Forced Prolonged Position (FPP)—both of which require accurate identification of the affected side, as they are designed to act on a single posterior semicircular canal and to mobilize otoconia located close to the PSC common crus. These techniques are effective in either resolving the TDB-PPN, or transforming it into TUB-PPN typical of geotropic PSC-BPPV. Conversely, in true contralateral ASC-BPPV, they are ineffective in directing debris toward the common crus, owing to the steep and vertical orientation of the non-ampullary arm of the anterior canal. Similar concepts have been previously described in the literature under different denominations, such as the “Bascule maneuver” and the “Reverse Semont maneuver”, all sharing the common biomechanical objective of mobilizing otoconial debris from atypical or less accessible segments of the posterior semicircular canal, particularly near the non-ampullary arm or the common crus. The present study builds upon this conceptual framework, proposing a maneuver specifically adapted to the vertical canal planes.

Both diagnosis and treatment become even more challenging when the torsional component of the nystagmus cannot be reliably assessed, resulting in the observation of a purely down-beating positional nystagmus. In such cases, it was considered necessary to develop a maneuver capable of achieving high success rates even when the affected side cannot be easily determined. Such a maneuver would take advantage of the Left Anterior–Right Posterior (LARP) and Right Anterior–Left Posterior (RALP) planes, along which the vertical semicircular canals are oriented, to mobilize otoconial debris regardless of precise lateralization. The objective of our study was to evaluate the ability of the Bascule/Pendular maneuver to convert the apogeotropic variant of posterior canal BPPV into the geotropic form, thereby enabling treatment with standard repositioning maneuvers.

## 2. Materials and Methods

This study was conducted on a sample of 178 patients, including 107 women (60.1%) and 71 men (39.9%), aged between 18 and 83 years (mean age: 55.2 years). Patients were recruited over a 10-year period, from November 2015 to November 2025, at the Otoneurology Center of Matera, Basilicata, Italy (Table 1).

All enrolled subjects had a positive history of symptomatic vertigo, with symptom onset between 4 and 7 weeks prior to evaluation (mean duration: 5 weeks). A total of 65.2% of patients reported a previous episode of benign paroxysmal positional vertigo (BPPV), whereas 34.8% presented with post-traumatic forms, including craniofacial surgery within the previous three months or head injury within the last 30 days.

All patients underwent a complete audiometric and tympanometric evaluation.

Patients with a clinical history of otoneurological disorders other than BPPV—such as migraine, vestibular neuritis, or Ménière’s disease—were excluded from the study.

Central causes of down-beating nystagmus were excluded based on a comprehensive clinical and neuro-otological assessment. Specifically, patients presenting with neurological red flags—such as persistent spontaneous down-beating nystagmus independent of head position, focal neurological deficits, severe headache, diplopia, dysarthria, limb ataxia, or gait instability—were not included. Moreover, all enrolled patients exhibited positional, transient, and fatigable nystagmus strictly related to positional maneuvers, with no accompanying neurological signs, supporting a peripheral vestibular origin. Where clinically indicated, patients had previously undergone neuroimaging as part of routine care, with no evidence of central pathology.

All enrolled patients were evaluated for positional nystagmus using a standardized diagnostic protocol [14], performed under video-Frenzel goggle monitoring. Notably, all 178 patients included in the study exhibited a pure down-beating positional nystagmus, without any torsional component. The diagnostic battery comprised the following tests:

- Upright Head Pitch Test (HPT)

The patient’s head was slowly flexed 60° forward and extended 30° backward relative to the horizontal plane while sitting. The examiner assessed the presence of vertical nystagmus, with or without torsional components.

- uRALP and uLARP tests

These tests were performed in the sitting position by rotating the patient’s head 45° to the left (uRALP) or 45° to the right (uLARP), followed by slow 60° forward flexion and 30° backward extension along the corresponding vertical canal plane. The presence of vertical nystagmus, with or without torsional components, compatible with posterior semicircular canal excitation (during extension) or inhibition (during flexion), was evaluated.

- Dix–Hallpike Test (DHT)

The test was performed by rapidly moving the patient from a seated position to a supine head-hanging position, with the head turned 45° toward the side being tested and extended approximately 20–30° below the horizontal plane, thereby aligning the posterior semicircular canal with the sagittal plane of motion. Latency, direction, duration, and fatigability of the induced vertical-torsional nystagmus were recorded. The test was initially performed on the suspected side and, if negative or inconclusive, repeated on the contralateral side.

**Figure 1:**
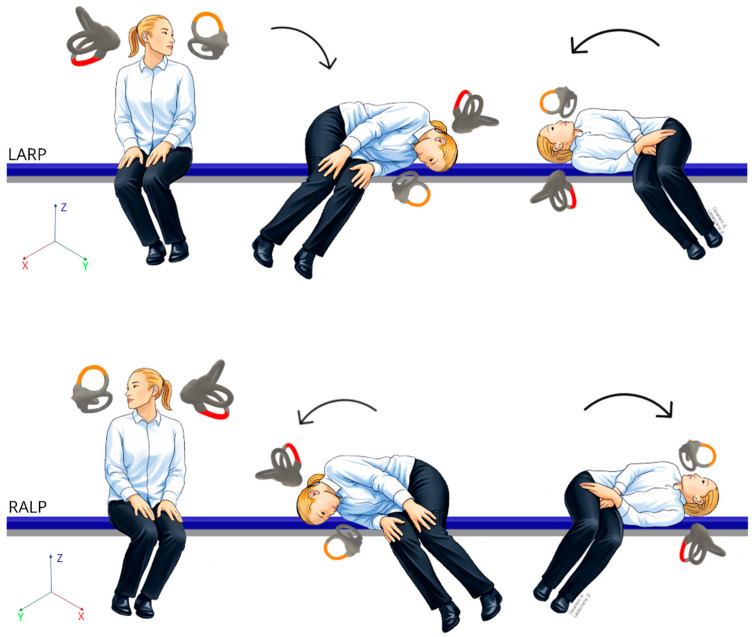
The Bascule/Pendular maneuver performed in the LARP and RALP.

All patients subsequently underwent the Bascule/Pendular maneuver (Figure 1), a theoretical modification of the classical Semont maneuver for posterior semicircular canal BPPV.

The procedure was initiated with the patient seated on the examination bed, with the head rotated 45° toward the side opposite to that in which the patient reported more intense vertigo and/or in which the Dix–Hallpike maneuver elicited the more intense down-beating nystagmus.

The patient was then gently brought into a lateral decubitus position on the corresponding side, aligning the head–trunk unit with the LARP, with the nose oriented downward, in a configuration analogous to a demi-Semont maneuver. Subsequently, the patient was rapidly swung to the contralateral side through a 180° rotation along the same plane, resulting in a nose-up head position.

When the affected side was correctly identified, geotropization of the nystagmus was observed, enabling the subsequent execution of a conventional liberatory maneuver for geotropic posterior semicircular canal BPPV (Semont maneuver). In cases in which the affected side could not be definitively identified, the Bascule/Pendular maneuver was repeated on the contralateral plane (RALP), following a sequence symmetrical to that described above. In our retrospective study, we included patients presenting with downbeating positional nystagmus who subsequently converted to a typical geotropic nystagmus of the posterior semicircular canal.

## 3. Results

The Bascule/Pendular maneuver was well tolerated, and no procedural interruptions or complications were recorded (Table 2).

In 86 patients (48.3%), immediate conversion of nystagmus from the apogeotropic to the geotropic variant was achieved following a single Bascule/Pendular maneuver, owing to correct identification of the affected side at the first attempt. In the remaining 92 patients (51.7%), geotropization of the nystagmus was achieved after additional Bascule/Pendular maneuvers. Specifically, 78 patients (43.8% of the total cohort) showed successful conversion to the geotropic pattern following a second Bascule/Pendular maneuver performed on the contralateral side, whereas 14 patients (7.9%) required more than two Bascule/Pendular maneuvers along the LARP and RALP before successful conversion was observed. Once geotropization had been achieved, the Semont maneuver [15] was systematically performed in all cases. The majority of patients reported complete resolution of positional vertigo within days. No early recurrences were observed at short-term follow-up (within 4–6 weeks), in line with routine clinical reassessment at our center.

## 4. Discussion

The morphological characteristics of the pure down-beating nystagmus observed in our selected patients treated with the Bascule/Pendular maneuver suggest two possible underlying mechanisms.

On one hand, the findings are compatible with apogeotropic PSC-BPPV, with otoconial particles floating within the distal portion of the non-ampullary arm near the common crus. On the other hand, the same nystagmus pattern could reflect ASC-BPPV of the contralateral side, with debris located in the ampullary arm.

In cases in which the observed pure downbeat nystagmus was attributable to an apogeotropic variant of the PSC on the same side, the Bascule/Pendular maneuver converted short-arm nystagmus into the more typical periampullary long-arm posterior canal nystagmus in approximately half of the patients. Following this conversion, a conventional posterior canal canalith repositioning maneuver was performed.

Similar maneuvers to the Bascule/Pendular maneuver have been described previously in the literature under different names, including the ‘Bascule maneuver’ [16] and the ‘Reverse Semont maneuver’ [17,18]. Despite differences in nomenclature and conceptual frameworks, all these techniques share the primary goal of mobilizing otoconia located in atypical or less accessible segments of the posterior canal, particularly near the non-ampullary arm or the common crus [19].

Our findings indicate that, in patients with suspected apogeotropic variants of ipsilateral PSC-BPPV, the Bascule/Pendular maneuver facilitated immediate transformation to the typical geotropic form in roughly 50% of cases, allowing prompt and effective treatment with standard posterior canal repositioning maneuvers. Owing to its simplicity of execution and high success rate, the Bascule/Pendular maneuver should be considered a first-line approach in patients presenting with pure down-beating positional nystagmus and a strong clinical suspicion of apogeotropic posterior semicircular canal involvement. Despite potential musculoskeletal limitations in elderly patients, the Bascule/Pendular maneuver was feasible and well tolerated in our cohort. Our results support the use of targeted head-plane maneuvers as an initial step to clarify canal involvement and optimize subsequent therapeutic outcomes [20,21].

### Study Limitations

This study has several limitations that should be acknowledged. First, its single-center design may limit the generalizability of the findings to other clinical settings with different patient populations or diagnostic practices. Second, the absence of a control group or a comparative repositioning maneuver prevents direct evaluation of the relative efficacy of the Bascule/Pendular maneuver against established therapeutic approaches. In addition, the observational nature of the study does not allow for causal inferences regarding treatment effectiveness. Furthermore, due to the retrospective design of the study, the exact number of patients who underwent neuroimaging (MRI/CT) could not be reliably retrieved, which may limit the completeness of the reported diagnostic data. Finally, the lack of systematic long-term follow-up represents an additional limitation, as recurrence rates and long-term symptom control could not be assessed. Future prospective, controlled, and multicenter studies with standardized follow-up protocols are therefore warranted to confirm these results and to better define the role of the Bascule/Pendular maneuver in the management of atypical BPPV presentations.

## 5. Conclusions

BPPV is primarily a mechanical disorder of the peripheral vestibular system, most commonly affecting the posterior semicircular canal. Atypical apogeotropic variants of posterior semicircular canal BPPV (PSC-BPPV) can mimic contralateral anterior semicircular canal (ASC) involvement, creating substantial diagnostic uncertainty. The Bascule/Pendular maneuver provides a practical and effective approach for mobilizing otoconial debris in less accessible portions of the posterior canal, converting apogeotropic forms into the more readily treatable geotropic variant, and thereby facilitating management with standard canalith repositioning maneuvers. Further prospective studies are required to validate its efficacy, optimize procedural protocols, and define its role within the diagnostic and therapeutic algorithm for atypical BPPV presentations.

## Figures and Tables

**Table 1:** Demographic and Clinical Characteristics of the Study Population (*n* = 178).

Number of patients	178
Sex, *n* (%)	
-Female	107 (60.1%)
-Male	71 (39.9%)
Age (years)	
-Range	18–83
-Mean ± SD	55.2 ± 14.6
Symptom duration before evaluation	
-Range	4–7 weeks
-Mean	5 weeks
Previous history of BPPV, *n* (%)	116 (65.2%)
Post-traumatic BPPV, *n* (%)	62 (34.8%)

**Table 2:** Outcomes of the Bascule–Pendular Maneuver.

Outcome	*n* (%)
Geotropization at the first Bascule–Pendular maneuver	86 (48.3%)
Geotropization at the second Bascule–Pendular maneuver	78 (43.8%)
-Multiple repetitions required	14 (7.9%)
Plane required for successful geotropization	
-LARP	97 (54.5%)
-RALP	81 (45.5%)

## Data Availability

The original contributions presented in this study are included in the article. Further inquiries can be directed to the corresponding author.
